# Effects of Bacillus Subtilis-Fermented White Sword Bean Extract on Adipogenesis and Lipolysis of 3T3-L1 Adipocytes

**DOI:** 10.3390/foods10061423

**Published:** 2021-06-19

**Authors:** Yujeong Choi, Da-Som Kim, Min-Chul Lee, Seulgi Park, Joo-Won Lee, Ae-Son Om

**Affiliations:** 1 Department of Food and Nutrition, College of Human Ecology, Hanyang University, Seoul 04763, Korea; choiyujeong@hanyang.ac.kr (Y.C.); dsom8383@hanyang.ac.kr (D.-S.K.); whi0314@hanyang.ac.kr (M.-C.L.); tmfrl13@hanyang.ac.kr (S.P.); j0131j@hanyang.ac.kr (J.-W.L.); 2 Department of Active Aging Industry, Division of Industrial Information Studies, Hanyang University, Seoul 04763, Korea

**Keywords:** *Canavalia gladiata*, triglyceride, glycerol, AMP-activated protein kinase, peroxisome proliferator-activated receptor

## Abstract

To investigate the adipogenesis and lipolysis effects of the Bacillus subtilis-fermented white sword bean extract (FWSBE) on 3T3-L1 adipocytes, we treated 3T3-L1 preadipocytes before and after differentiation with FWSBE and measured triglyceride, free glycerol, mRNA, and protein levels. First, FWSBE reduced the cell viability of 3T3-L1 pre-adipocytes under 1000 µg/mL conditions. Triglyceride accumulation in 3T3-L1 pre-adipocytes was suppressed, and free glycerol content in mature 3T3-L1 adipocytes was increased in the FWSBE treatment groups, indicating that FWSBE has anti-obesity effects. Further, FWSBE suppressed adipogenesis in 3T3-L1 pre-adipocytes by lowering the protein levels of C/EBPα, PPARγ, and FAS and increasing the level of pACC and pAMPK. Additionally, FWSBE promoted lipolysis in mature 3T3-L1 adipocytes by increasing the transcription levels of Ppara, Acox, and Lcad and the protein levels of pHSL and ATGL. Thus, we suggest that FWSBE can be a potential dietary supplement because of its anti-obesity properties.

## 1. Introduction

Obesity is associated with various metabolic complications, such as type 2 diabetes, cardiovascular disease, high blood pressure, and dyslipidemia. The growing prevalence of obesity is a public health concern among many modern societies [1,2]. In general, obesity is caused when excessive energy is accumulated in white adipose tissue in the form of triglyceride (TG), which is composed of three fatty acids and glycerol [3]. Adipogenesis is the process of differentiation from pre-adipocytes to adipocytes caused by the stepwise action of adipogenic transcription factors. The three elements mainly involved are CCAAT-enhancer-binding proteins (C/EBPs), peroxisome proliferator-activated receptors (PPARs), and sterol regulatory element-binding proteins (SREBPs) [4,5]. Among these elements, C/EBPα and PPARγ control the levels of adipocyte fatty acid binding protein (aP2), adiponectin, acetyl-CoA carboxylase (ACC), and fatty acid synthase (FAS). These determine the actual adipocyte phenotype and intracellular lipid accumulation [6,7]. However, when the ratio of AMP/ATP increases, AMP-activated protein kinase (AMPK) is activated and regulates the energy balance of cells by inhibiting lipogenesis and promoting lipolysis [8]. Additionally, the lipid droplet (LD), which is the major storage organelle of TG, is degraded by enzymes such as hormone-sensitive lipase (HSL) and adipose triglyceride lipase (ATGL) during lipolysis [9,10]. Thus, the released free glycerol and free fatty acids (FFAs) due to the breakdown of TG are important biomarkers that represent a decrease in adipocyte lipid [11]. PPARα plays a major role as a transcription factor that promotes the expression of FA oxidation genes, such as peroxisomal acyl-coenzyme A oxidase 1 (ACOX1), long-chain acyl-CoA dehydrogenase (LCAD), and medium-chain acyl-CoA dehydrogenase (MCAD) [12,13].

Since obesity is considered a key health concern in developed countries, researches are developing anti-obesity drugs (e.g., phentermine, phendimetrazine, and lorcaserin) [14]. However, anti-obesity drugs can cause several side effects, such as insomnia, headaches, and constipation [15,16]. Therefore, in recent years, interest in natural substances with safety and functionality has increased [17]. Among the natural materials, soybeans have drawn substantial attention since they have been reported to contain various bioactive compounds, including tocopherols, soyasaponins, and isoflavones [18,19]. These functional substances not only improve metabolic and cardiovascular functions but also help to prevent obesity [20,21,22,23].

The sword bean (Canavalia gladiata) belonging to the legume family is cultivated mainly in tropical and subtropical regions of Asia [24]. In Korea and Japan, the sword bean was used in folk remedies for the treatment of purulent inflammation. Sword beans have also been effective as antioxidants [25] and have anti-cancer [26], antibacterial [27], anti-diabetic [28], and anti-gastritis [29] properties. In addition, the sword bean has a significantly lower fat content (1.2 ± 0.13%) than the soybean (16.5 ± 0.29%) and black soybean (16.1 ± 0.15%), and the total flavonoid content is significantly higher (493.2 ± 21.2 mg/100 g) in sword beans than in soybeans (71.8 ± 6.3 mg/100 g) and black soybeans (97.5 ± 14.9 mg/100 g) [30]. However, research on the anti-obesity effects of sword beans is still limited.

In this study, to investigate the effect of Bacillus subtilis-fermented white sword bean extract (FWSBE) on adipogenesis and lipolysis of 3T3-L1 adipocytes, FWSBE was used to treat adipocytes before and after differentiation. TG, cell differentiation, and adipogenesis biomarkers were measured in the premature adipocyte, and free glycerol and lipolysis biomarkers were measured in the mature adipocyte.

## 2. Materials and Methods

### 2.1. Sample Preparation and Proximate Analysis

B. subtilis-fermented white sword bean extract (FWSBE) was provided by the Korea Food Research Institute (KFRI, Wanju-gun, Jeollabuk-do, Korea). The general ingredients of FWSBE were ascertained by following the general test method of the Korean Food Standards Codex [31]. The composition of freeze-dried powder samples of the fermented white sword bean was 24.09% soluble dietary fiber, 13.26% carbohydrate, 60.32% crude protein, 1.79% crude fat, and 6.88% crude ash. 

### 2.2. Cell Culture and Differentiation 

The cell line used in this study was 3T3-L1, a pre-adipocyte cell line, distributed from Korean Cell Line Bank (KCLB, Jongno, Seoul, Korea). Cells were cultured in a 5% CO2 incubator at 37 °C using Dulbecco’s Modified Eagle’s Media (DMEM; WELGENE, Gyeongsan, Gyeongsanbuk-do, Korea), with 10% bovine calf serum (BCS; Thermo Fisher Scientific, Waltham, MA, USA) and 1% penicillin-streptomycin (PS; WELGENE, Gyeongsan, Gyeongsanbuk-do, Korea). Cells were dispensed into a 6-well plate at a concentration of 3 × 104 cells/mL. Changing the media every 2 days, the cells were cultured to 100% and then cultured for 2 days to achieve a post-confluent state (day 0). After that, MDI solution containing 1 µM dexamethasone (DEX), 0.5 mM 3-isobutyl−1-methylxanthine (IBMX), 10 µg/mL insulin (Sigma-Aldrich Co., St. Louis, MO, USA), and 10% fetal bovine serum (FBS; Sigma-Aldrich Co., St. Louis, MO, USA) and DMEM containing 1% PS was used to treat cells for 2 days to induce cell differentiation (days 1 and 2). Next, to promote differentiation, DMEM containing 10 µg/mL insulin, 10% FBS, and 1% PS was changed every 48 h for a total of 6 days (days 3 to 8). Cells were then treated with DMEM containing 10% FBS and 1% PS for an additional 6 days (days 9 to 14). To confirm the inhibitory effect of FWSBE on adipocyte differentiation, cells were treated with FWSBE and MDI for 48 h (days 1 and 2) at each of the four concentrations (100, 200, 400, and 1000 μg/mL). Additionally, to check the lipolysis and FA oxidation effects of FWSBE, the mature 3T3-L1 adipocytes were treated at each of the four concentrations every 48 h for a total of 96 h after differentiation was completed (days 15 to 18). All experiments were performed in triplicate.

### 2.3. Cell Viability Assay

The cytotoxicity of the FWSBE in 3T3-L1 cells was evaluated. 3T3-L1 pre-adipocytes were dispensed into a 12-well plate at a concentration of 3 × 104 cells/mL and then incubated for 24 h in DMEM containing 10% BCS. After 24 h, the culture solution was removed and each of the four concentrations of FWSBE (100, 200, 400, and 1000 μg/mL) was used to treat cells, which were then incubated for 24 h. Cells were then washed with PBS and DMEM-MTT reagent (1:9) and were incubated for 4 h in a 5% CO_2_ incubator at 37 °C to generate formazan. Centrifugation (LABOGENE 1248, multi-purpose centrifuge set, LABOGENE, Seoul, South Korea) at 4 °C at 3000 rpm for 3 min followed, and 1 mL of DMSO was added to each well to dissolve the produced formazan. Absorbance was measured at a wavelength of 540 nm using an ELISA microplate reader (Thermo Fisher Scientific, Waltham, MA, USA). All experiments were performed in triplicate.

### 2.4. Quantification of Triglyceride Content

AdipoRed™ assay reagent (LONZA, Walkersville, MD, USA) was used for measuring the TG content and was performed according to the manufacturer’s instructions. Cells were treated with FWSBE and MDI for the first 48 h (days 1 and 2) at each of four concentrations (100, 200, 400, and 1000 μg/mL) and were allowed to differentiate for 14 days. After removing the culture medium and washing with PBS, each well was treated with 2 mL of PBS and 60 μL of AdipoRed at room temperature for 10 min. Thereafter, fluorescence was measured at excitation 485 nm and emission 590 nm wavelengths using a fluorescence spectrophotometer (Synergy™ HTX Multi-Mode Microplate Reader, BioTek, Sinooski, VT, USA). All experiments were performed in triplicate.

### 2.5. Quantification of Free Glycerol Content

To measure the effect of FWSBE on lipolysis, free glycerol was measured according to the manufacturer’s instructions using a cell-based glycerol assay kit (Cayman Chemical, Ann Arbor, MI, USA). After completing differentiation at a concentration of 3 × 104 cells/mL, the mature 3T3-L1 adipocytes were treated at each of the four concentrations every 48 h for a total of 96 h after differentiation was completed (days 15 to 18). The supernatant of the medium was collected after 96 h. To a new 96-well plate, 25 µL of glycerol standards for each concentration and 25 µL of the supernatant from the control and FWSBE wells were dispensed. Then, 100 µL of free glycerol assay reagent was added to each well. After incubation at room temperature for 15 min, absorbance was measured at 540 nm. All experiments were performed in triplicate.

### 2.6. Quantitative RT-PCR Analysis

Total RNA was extracted with TRIzol™ reagent (Invitrogen, Carlsbad, CA, USA), according to the manufacturer’s instructions. Quantity and purity were analyzed spectrophotometrically at 230, 260, and 280 nm (QIAxpert, Qiagen, Hilden, Germany). To synthesize cDNA using a quantitative real-time reverse transcription-polymerase chain reaction (qRT-PCR), one microgram of total RNA and oligo (dT)20 primer were used (SuperScript™ II RT kit, Invitrogen, Carlsbad, CA, USA). For real-time RT-PCR amplification, ach reaction consisted of 1 μL of cDNA that had been reverse transcribed from 1 μg of total RNA and 0.2 μM of real-time RT-F/R. qRT-PCR was conducted at 95 °C for 4 min then 40 cycles of 95 °C for 10 s, 58 °C for 30 s, 72 °C for 1 min, and 72 °C for 10 min using SYBR Green as a probe (Molecular Probes Inc., Eugene, OR, USA) and a CFX96™ real-time PCR system (Bio-Rad, Hercules, CA, USA). To confirm the amplification of specific products, melting curve cycles were conducted using the settings 95 °C for 1 min, 55 °C for 1 min, and 80 cycles of 55 °C for 10 s with a 0.5 °C increase per cycle using qRT-PCR forward or reverse primers (Table 1). β-actin gene expression, which was stable throughout the experiments, was used as an internal control to normalize expression levels between samples. All experiments were performed in technical triplicates. A relative fold-change in gene expression compared to the control was calculated by the 2^−ΔΔCt^ comparative method [32].

### 2.7. Western Blot Analysis

After removing the culture medium from 3T3-L1 cells, the cells were washed with PBS and RIPA lysis buffer (Thermo Fisher Scientific, Waltham, MA, USA) containing protease inhibitor and phosphatase inhibitor (Sigma-Aldrich Co., St. Louis, MO, USA) and were dispensed to isolate the protein. The supernatant was then obtained after centrifugation (LABOGENE 1248, multi-purpose centrifuge set, LABOGENE, Seoul, South Korea) at 4 °C at 13,000 rpm for 20 min. Protein concentration was quantified using a BCA protein assay kit (Thermo Fisher Scientific, Waltham, MA, USA). Samples for Western blot analysis were prepared by adding samples, distilled water, and 4X LaemmLi Sample Buffer (Bio-rad, Hercules, CA, USA) according to the protein quantification level. The quantified protein was subjected to electrophoresis using 8–12% SDS-polyacrylamide gel (SDS-PAGE) and then transferred using polyvinylidene fluoride (PVDF) membranes (Sigma-Aldrich Co., St. Louis, MO, USA). After blocking with 5% skim milk for 1 h at room temperature, the primary antibodies were added. The membranes were held overnight at 4 °C (Table 2). Secondary antibody reactions occurred during processing at room temperature for 1 h. ECL detection reagents (Bio-rad, Hercules, CA, USA) were applied to the membrane, and the expression levels of the protein were confirmed using ChemiDoc (Bio-Rad, Hercules, CA, USA). Each protein band was quantified using ImageJ (NIH, Bethesda, MD, USA). All experiments were performed in triplicate.

### 2.8. Statistical Analysis

For statistical analysis, SPSS version 18.0 (SPSS Inc., Chicago, IL, USA) was used, and the data are presented as mean ± standard deviation (S.D.). One-way ANOVA was used to analyze the significant differences between the control and test groups; this was followed by Duncan’s test. The differences with *p* < 0.05 were considered significant.

## 3. Results 

### 3.1. Effects of FWSBE on Cell Viability in 3T3-L1 Preadipocytes

**Figure 1:**
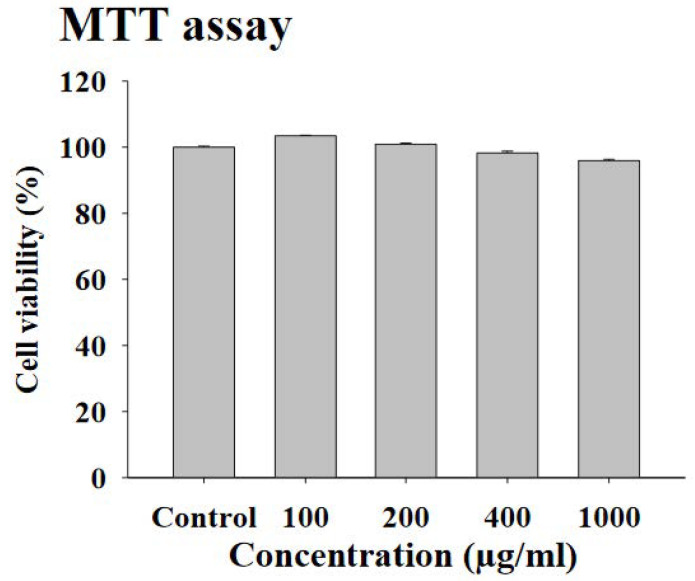
Effects of fermented white sword bean extract (FWSBE) on cell viability of 3T3-L1 preadipocytes. The 3T3-L1 cells (5 × 10^4^ cells/mL) were treated with FWSBE at various concentrations (100, 200, 400, and 1000 μg/mL) for 24 h. Cell viability was measured by 3-(4,5-Dimethylthiazol-2-yl)-2,5-diphenyltetrazolium bromide (MTT) assay. Values are presented as mean ± standard deviation.

To measure the cell viability, 3T3-L1 preadipocytes were exposed to each of the four concentrations (100, 200, 400, and 1000 μg/mL) of FWSBE for 24 h and an MTT assay was conducted. Every concentration showed a survival rate higher than 99%, indicating that no cytotoxicity was induced by FWSBE (Figure 1). 

### 3.2. Effects of FWSBE on Triglyceride and Free Glycerol Content

**Figure 2:**
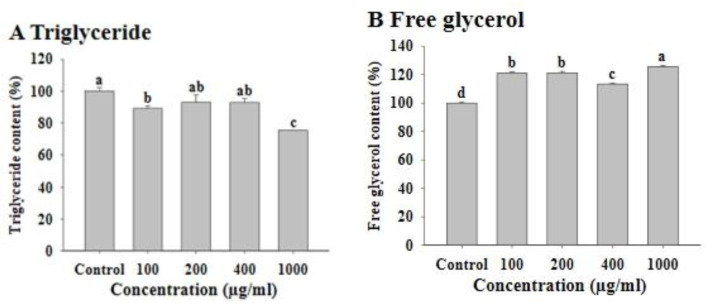
(**A**) Effects of fermented white sword bean extract (FWSBE) on triglyceride accumulation in 3T3-L1 pre-adipocytes. The cells were treated with FWSBE at various concentrations (100, 200, 400, and 1000 μg/mL) for 48 h during differentiation. The cumulative triglyceride content was measured with the AdipoRed assay. (**B**) Effects of FWSBE on free glycerol in mature 3T3-L1 adipocytes. 3T3-L1 cells (3 × 104 cells/mL) were exposed to different concentrations (100, 200, 400, and 1000 μg/mL) of FWSBE for 96 h. Values are presented as mean ± standard deviation. The statistically significant difference (*p* < 0.05) between groups was determined by a one-way ANOVA and Duncan’s multiple range test. Different letters are indicated as significant differences.

To investigate the effect of FWSBE on adipogenesis, 3T3-L1 preadipocytes were exposed to each of the four concentrations of FWSBE (100, 200, 400, and 1000 μg/mL) for the first 48 h with MDI and were allowed to differentiate for 14 days. In the FWSBE-treated groups, the intracellular TG contents significantly decreased in 100 (approximately 10.5%) and 1000 (approximately 24.4%) μg/mL exposure groups (*p* < 0.05) (Figure 2A; Figure S1). Next, to measure the effect of FWSBE on lipolysis, mature 3T3-L1 adipocytes were exposed to each concentration of FWSBE for 96 h, and the content of free glycerol in the medium was measured. The amount of free glycerol was significantly (*p* < 0.05) increased to 120.95% (100 μg/mL), 121.22% (200 μg/mL), 113.42% (400 μg/mL), and 125.57% (1000 μg/mL) compared to the control (Figure 2B). 

### 3.3. Effects of FWSBE on the Adipogenesis in 3T3-L1 Pre-Adipocytes 

**Figure 3:**
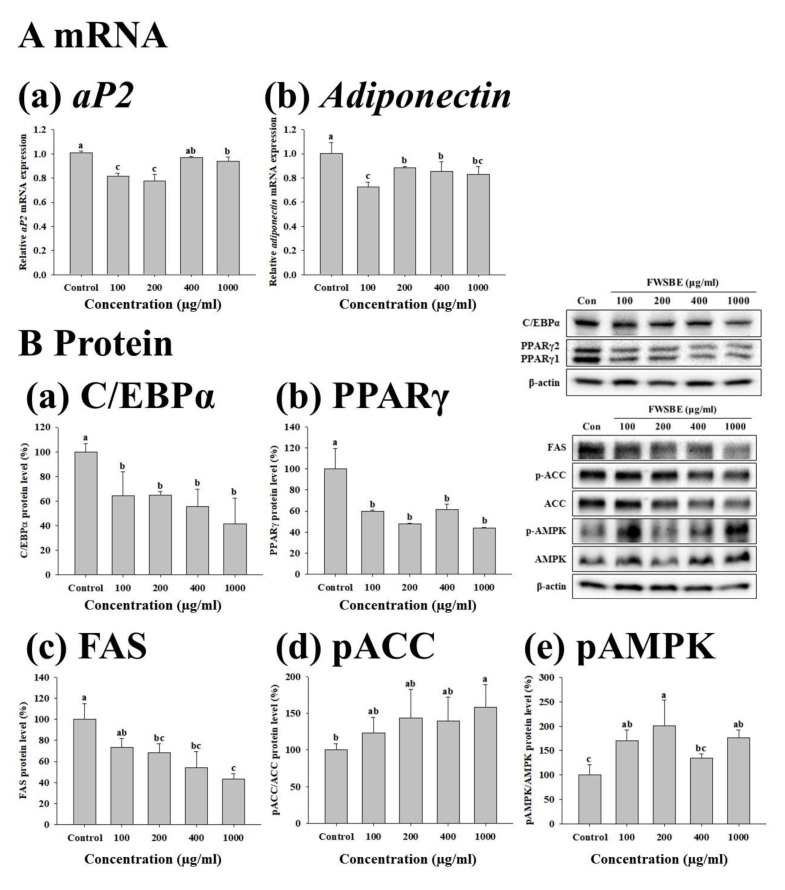
Effects of fermented white sword bean extract (FWSBE) on (**A**) the transcription level of (a) aP2 and (b) adiponectin and (**B**) the protein level of (a) C/EBPα, (b) PPARγ, (c) FAS, (d) pACC, and (e) pAMPK on 3T3-L1 preadipocytes. The cells were treated with FWSBE at various concentration (100, 200, 400, and 1000 μg/mL) for 48 h during differentiation. Values are presented as mean ± standard deviation. The statistically significant difference (*p* < 0.05) between groups was determined by a one-way ANOVA and Duncan’s multiple range test. Different letters are used to indicate significant differences. C/EBPα, CCAAT/enhancer binding protein α; FAS, fatty acid synthase; ACC, acetyl-coenzyme A carboxylase; PPARγ, peroxisome proliferator-activated receptor γ; and AMPK, AMP-activated protein kinase.

To investigate the effects of FWSBE (100, 200, 400, and 1000 μg/mL) on adipogenesis, we treated 3T3-L1 preadipocytes with FWSBE for 48 h, measuring mRNA transcription and protein levels. In adipogenesis-specific gene transcription, the transcript level of aP2 and adiponectin significantly (*p* < 0.05) decreased compared to the control, except for aP2 at 400 μg/mL of FWSBE treatment (Figure 3A). Additionally, the protein levels of C/EBPα, PPARγ, and FAS were significantly (*p* < 0.05) decreased, and the protein levels of pACC and pAMPK significantly (*p* < 0.05) increased compared to the control (Figure 3B).

### 3.4. Effects of FWSBE on Lipolysis in the Mature 3T3-L1 Adipocytes

**Figure 4:**
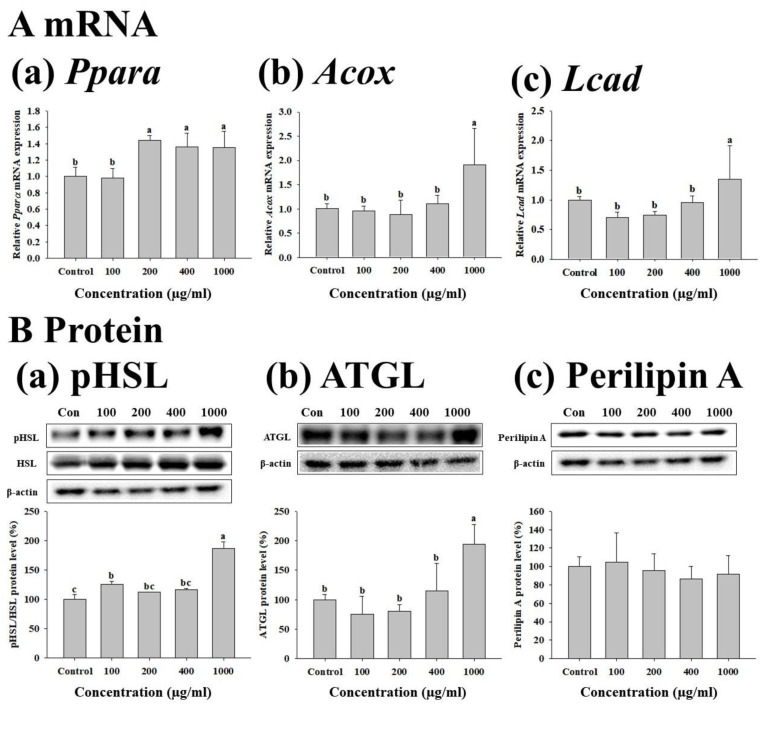
Effects of fermented white sword bean extract (FWSBE) on (**A**) transcription level of (a) *Ppara*, (b) *Acox*, and (c) *Lcad*, and (**B**) the protein level of (a) pHSL, (b) ATGL, and (c) perilipin A in mature 3T3-L1 adipocytes. The cells were treated with FWSBE at various concentrations (100, 200, 400, and 1000 μg/mL) for 96 h. Values are presented as mean ± standard deviation. The statistically significant differences (*p* < 0.05) between groups were determined by a one-way ANOVA and Duncan’s multiple range test. Different letters are used to indicate significant differences. Acox, acyl-coenzyme A oxidase; ATGL, adipose triglyceride lipase; HSL, hormone sensitive lipase; Lcad, long-chain acyl-coenzyme A dehydrogenase; and Ppara, peroxisome proliferator-activated receptor α.

To confirm the effect of the four concentrations of FWSBE (100, 200, 400, and 1000 μg/mL) on lipolysis, we measured the mRNA transcription and protein levels in mature 3T3-L1 adipocytes after 96 h exposure to FWSBE. The transcript levels of Ppara, Acox, and LCAD were significantly (*p* < 0.05) decreased compared to the control at 1000 μg/mL (Figure 4A). To test whether FWSBE promoted TG decomposition in 3T3-L1 adipocytes, the protein levels of pHSL, ATGL, and perilipin A were measured. Both pHSL and ATGL showed a significant (*p* < 0.05) increase at 1000 μg/mL, but perilipin A did not show any changes in experimental groups (Figure 4B). 

## 4. Discussion

Obesity is a state in which body fat is over-accumulated in the body, which not only affects the quality of life but is also a public health concern due to the occurrence of chronic degenerative diseases and metabolic complications. Therefore, to reduce obesity, anti-obesity studies using natural substances are attracting attention [33,34,35]. Previous studies have reported that various phytochemicals present in natural substances can prevent obesity through the inhibition of oxidative stress and inflammation [36,37]. Foods with antioxidant activity may help to decrease body weight and obesity-related disorders [38,39]. In general, fermentation can increase the content of functional substances [25,40] and bioaccessibility due to changes in the physiological characteristics of active substances [41]. A previous study analyzed the composition of phytochemicals in fermented and non-fermented sword beans [42]. The results showed that the total phenolic and flavonoid contents of fermented sword beans were higher than those of non-fermented sword beans, and among the fermented sword beans, fermented red sword beans showed the highest contents. As a result of antioxidant activity analysis, DPPH was similar among fermented sword bean groups; however, nitrate-scavenging activity was higher in fermented white sword beans compared to the others. Additionally, a previous study has reported that the total flavonoid content of sword beans was about 5–6 times higher than that of soybeans and black soybeans; and the antioxidant activity of sword beans was comparable to that of α-tocopherol [43], indicating that FWSBE has higher ROS-scavenging ability through hydrogen donation [44]. Therefore, we focused on the anti-obesity effects of FWSBE, since FWSBE has the strongest antioxidant activity among sword beans and its extracts, even in our study (Figure S2), and the MTT experiment showed that FWSBE did not reduce cell viability even at the highest concentration (1000 μg/mL). 

To evaluate whether FWSBE has anti-obesity effects, 3T3-L1 pre-adipocytes were treated with FWSBE for 48 h and the TG content was measured. The concentration of TG was significantly reduced by FWSBE at the highest concentration (1000 μg/mL). TG is mainly stored in LD, an inactive vesicle produced by adipogenesis surrounded by a phospholipid monolayer [45]. TG is an ester-linked form of one molecule of glycerol and three molecules of FFA. In general, TG is used as an important energy source for cells, but excess TG stored in adipose tissue causes obesity [46]. Therefore, the content of TG was mainly used to evaluate the anti-obesity effects of natural products in previous studies. For example, when 3T3-L1 adipocytes were treated with fermented soybean extract at 10, 50, and 100 μg/mL, TG significantly decreased at all concentrations compared to the control [47]. Additionally, when 3T3-L1 adipocytes were treated with fermented soybean and non-fermented soybean extracts at 50 μg/mL, the content of TG was significantly reduced in the fermented soybean extract group compared to the control [48]. However, non-fermented soybean extract did not induce any changes in TG content, indicating that the adipogenesis inhibitory effect may be increased during the fermentation process. In addition to the TG content, mature 3T3-L1 adipocytes were treated with FWSBE, and the content of free glycerol released from the cells was measured. The content of free glycerol was significantly increased in the FWSBE-treated group. The degree of free glycerol released to the cell medium can be used as a marker in anti-obesity studies since TG is decomposed into FAs and glycerol by lipolytic enzymes [49]. In a previous study on the anti-obesity effect of soymilk fermented with B. subtilis, the amount of released free glycerol increased by 43% in the experimental group [50]. Additionally, when comparing fermented and non-fermented soybean extract, free glycerol secretion was significantly increased compared to the control group [48]. Taken together, our data clearly showed that FWSBE has anti-obesity capacity in both premature and mature 3T3-L1 adipocytes.

To understand how FWSBE suppressed TG accumulation in 3T3-L1 pre-adipocytes, mRNA transcription and protein levels were measured. Results showed that the transcript levels of aP2 and adiponectin were downregulated. In the late stages of differentiation, adipocyte-specific genes are expressed, thereby allowing for differentiation into adipocytes. aP2 is a target gene of PPARγ and is specifically expressed in adipocytes [51], and its expression promotes fatty acid absorption into adipocytes [52]. Adiponectin is an adipocytokine secreted from adipocytes and plays an important role in maintaining insulin sensitivity and energy homeostasis [53]. Therefore, these genes are widely used as biomarkers in anti-obesity research. For example, in previous studies using blueberry peel extract [54] and onion peel extract [55], which are natural products rich in polyphenol content, the transcript level of adipocyte-specific genes decreased due to the decrease in the expression of adipogenic transcription factors. This suggests that those extracts have an anti-obesity effect. Conversely, overexpression of adiponectin in 3T3-L1 cells increased the expression of adipogenic transcription factors in the early and late stages of differentiation, suggesting that adipocyte-specific genes could regulate the adipogenesis process [56]. However, although adiponectin showed a decrease in adiponectin, which is one of the major biomarkers related to adipocyte differentiation, in blueberry peel extract [54] and onion peel extract [55], a decrease in adiponectin might cause side effects, such as type 2 diabetes, obesity, and cardiovascular disease in humans [57]. Therefore, further researches about FWSBE against side effects in humans are needed.

The process of adipogenesis, the differentiation from pre-adipocytes into adipocytes, is triggered by the stepwise regulation of many types of adipogenic transcription factors. Among the transcription factor products, AMPK is the key enzyme for regulating lipid metabolism. When the intracellular AMP/ATP ratio increases, AMPK is activated by phosphorylation of the threonine 172 residue of the α-subunit [58]. SREBP-1c, which is expressed by insulin in the early stages of differentiation, is an essential transcription factor for the synthesis of FAs and regulates the expression of C/EBPα and PPARγ [59]. C/EBPα and PPARγ are key regulators of adipogenesis that promote the post-differentiation process of adipocytes [60]. Lipogenesis is a process in which acetyl-CoA is converted to malonyl-CoA by the action of ACC in the cytoplasm, and FA is synthesized by the action of PPARγ-regulated FAS. Therefore, in general, when AMPK is activated in a specific situation (in this study, FWSBE treatment), activated AMPK suppresses the expression of SREBP-1c [61,62] to suppress PPARγ and the suppressed PPARγ inhibits the expression of the target genes for ACC and FAS [63,64,65]. Therefore, this mechanism is widely used in anti-obesity studies. For example, when 3T3-L1 cells were treated with bamboo leaf extract at 100 μg/mL [66] or siRNA of AMPK [67,68], the protein levels of C/EBPα, PPARγ, SREBP-1c, and FAS significantly decreased compared to the control. In addition, pACC and pAMPK protein expression increased, indicating that adipogenesis was suppressed. Additionally, AMPK was phosphorylated by soyasaponin Af and quercetin 3-O-glucoside from the kidney beans (Phaseolus vulgaris L.) [69], genistein and daidzein from the soybeans [70], vitexin from the mung beans (*Vigna radiata*. L.) [71], and theobromine from cocoa beans (Theobroma cacao) [72]. Our data suggest that FWSBE-activated AMPK in 3T3-L1 pre-adipocytes leads to suppression of adipogenesis through post-translational regulation of adipogenic factors. 

To confirm the effects of FWSBE on lipolysis in mature 3T3-L1 adipocytes, mRNA and protein levels were measured. Results showed that Ppara, Acox, and Lcad were upregulated in FWSBE treatment experimental groups. Ppara encodes a transcription factor involved in fatty acid oxidation and peroxisome metabolism and regulates the expression of genes related to β-oxidation, such as Acox1 and Lcad [73,74]. The expression of both of these genes results in enzymes that convert FFAs to 2-trans-enoyl-CoA [75]. Therefore, this mechanism was studied in previous research studies. For example, in rats fed the flavonoid naringenin, Ppara expression was significantly increased in liver tissue, resulting in a decrease in TG [76]. Additionally, the transcript level of Acox1 increased due to the upregulation of Ppara by treatment with the flavonoid 2.4–240 µM naringenin in hepatocytes [77]. In addition to the mRNA level, the protein levels of pHSL, ATGL, and perilipin A were observed. Results showed that pHSL and ATGL were significantly increased in the FWSBE-treated group (at 1000 μg/mL) compared to the control; perilipin A did not show any changes. When HSL is phosphorylated and transferred from the cytoplasm to the surface of the LD [78], pHSL promotes the decomposition of TG in the LD. In addition, perilipin A, which presents on the surface of LD, is phosphorylated by PKA and combined with pHSL to promote the decomposition of TG [79]. ATGL, which is involved in the first step in the breakdown of TG, is also affected and promotes lipolysis [80]. Therefore, in lipolysis studies, the above biomarkers are commonly used. For example, after treatment with the flavonoid myricetin in 3T3-L1 cells, the expression level of perilipin A significantly decreased compared to the control; however, HSL increased, leading to increases in lipolysis [81]. Taken together, our data suggest that FWSBE promoted lipolysis in mature 3T3-L1 adipocytes through activation of HSL and ATGL expression. 

## 5. Conclusions

In this study, we investigated the potential anti-obesity effects of FWSBE in both premature and mature 3T3-L1 adipocytes. In the early stage of adipocyte differentiation, FWSBE phosphorylated AMPK, which led to a decrease in the mRNA expression of aP2 and adiponectin, and the protein levels of C/EBPα, PPARγ, and FAS also decreased, resulting in suppression of TG accumulation. Additionally, in mature 3T3-L1 adipocytes, FWSBE increased the mRNA expression of Ppara, Acox, and Lcad and the protein levels of pHSL and ATGL, promoting lipolysis in mature 3T3-L1 adipocytes. Overall, we confirmed that FWSBE affected both adipogenesis and lipolysis in 3T3-L1 cells. However, some biomarkers (e.g., adiponectin) could induce a negative impact in humans. Further studies are needed to apply to human health. We suggest that this study provides basic information on the anti-obesity effects of FWSBE. 

## Figures and Tables

**Table 1:** The primer sequences used for qRT-PCR.

Target	Primer (5′→3′)	
*aP2*	forward	AAGGTGAAGAGCATCATAACCCT
	reverse	TCACGCCTTTCATAACACATTCC
*Adiponectin*	forward	GCCTGTCCCCATGAGTAC
	reverse	TCTTCGGCATGACTGGGC
*Ppara*	forward	ACGATGCTGTCCTCCTTGATG
	reverse	GCGTCTGACTCGGTCTTCTTG
*Acox1*	forward	GCACCTTCGAGGGGGAGAACA
	reverse	GCGCGAACAAGGTCGACAGAA
*Lcad*	forward	TCCGCCCGATGTTCTCATTC
	reverse	AGGGCCTGTGCAATTTGAGT
*β-actin*	forward	ACCCCAGCCATGTACGTAGC
	reverse	GTGTGGGTGACCCCGTCTC

**Table 2:** The primary antibodies used for Western blot analysis.

Target	Secondary Host	Size (kDa)	Dilution	Company	Catalog No.
C/EBPα	Rabbit	42	1:1000	Cell signaling Technology	#2295
PPARγ	Rabbit	53, 57	1:1000	Cell signaling Technology	#2443
p-AMPK	Rabbit	62	1:1000	Cell signaling Technology	#2531
AMPK	Rabbit	62	1:1000	Cell signaling Technology	#2532
p-ACC	Rabbit	280	1:1000	Cell signaling Technology	#3661
ACC	Rabbit	280	1:1000	Cell signaling Technology	#3676
FAS	Rabbit	273	1:1000	Cell signaling Technology	#3180
HSL	Rabbit	81, 83	1:1000	Cell signaling Technology	#4107
p-HSL	Rabbit	81, 83	1:1000	Cell signaling Technology	#4139
ATGL	Rabbit	54	1:1000	Cell signaling Technology	#2138
Perilipin A	Rabbit	62	1:1000	Cell signaling Technology	#9349
β-actin	Mouse	45	1:1000	Cell signaling	#3700

## Supplementary Materials

The following are available online at https://www.mdpi.com/article/10.3390/foods10061423/s1, Figure S1: Lipid droplet of FWSBE on the 3T3-L1 adipocytes. Cells were observed using microscope (X200 magnification; modified from Kim, 2020). Figure S2: DPPH free radical scavenging activity of white sword bean extract (WSBE) and fermented white sword bean extract (FWSBE). Values are presented as means ± standard deviation. Different letters indicate significant differences based on a one-way ANOVA and Duncan’s multiple range test (*p* < 0.05).
