# Supplementary material for: Effects of Bacillus Subtilis-Fermented White Sword Bean Extract on Adipogenesis and Lipolysis of 3T3-L1 Adipocytes
Source: Foods. 2021 Jun 19;10(6):1423. doi: 10.3390/foods10061423 (PMC8235212; doi:10.3390/foods10061423)
Supplement: Supplementary file 1 [file foods-10-01423-s001.zip › foods-1249164-supplementary.pptx]

## Slide 1
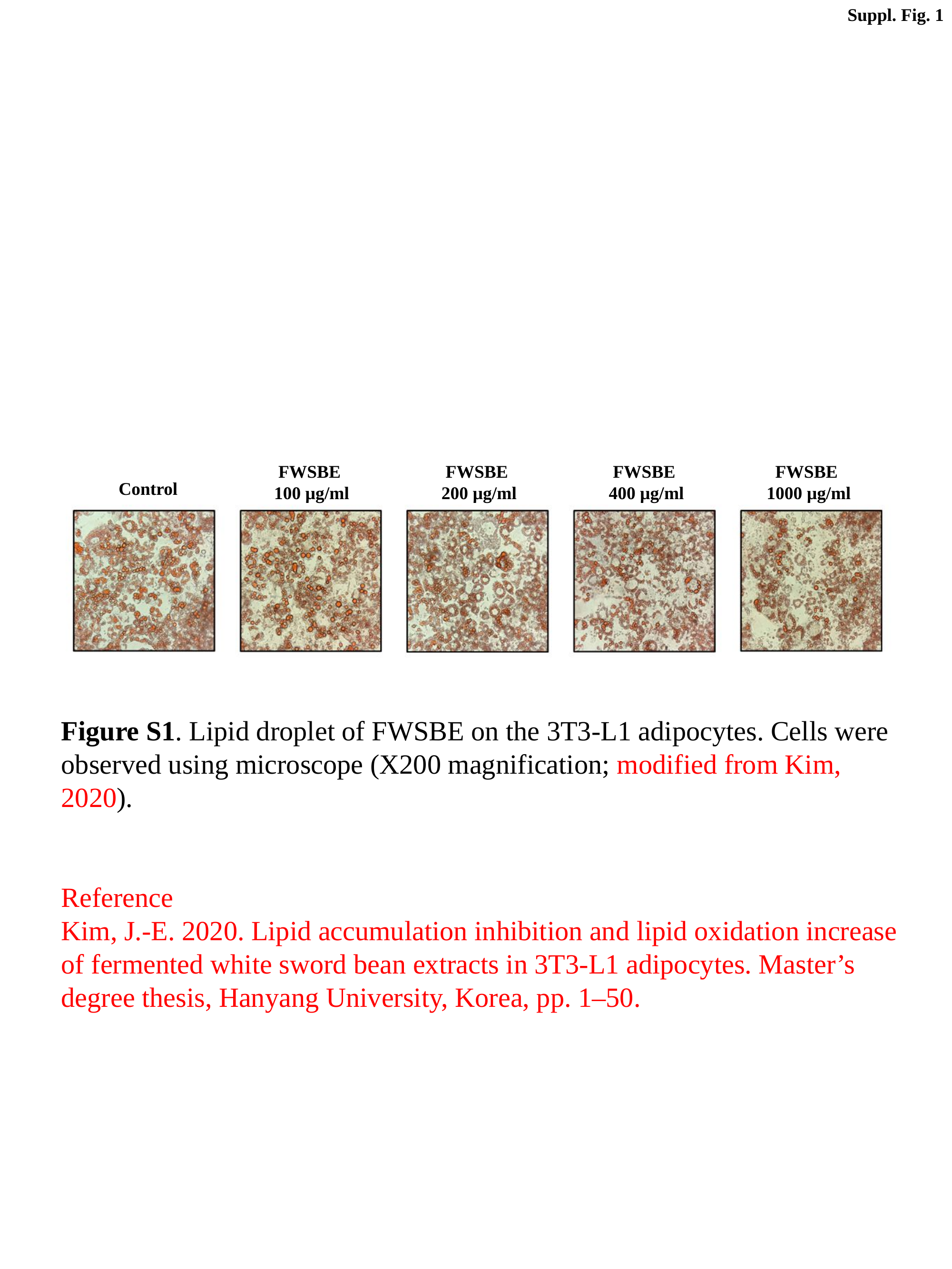

Suppl. Fig. 1
FWSBE
100 µg/ml
FWSBE
200 µg/ml
FWSBE
400 µg/ml
FWSBE
1000 µg/ml
Control
Figure S1. Lipid droplet of FWSBE on the 3T3-L1 adipocytes. Cells were observed using microscope (X200 magnification; modified from Kim, 2020).
Reference
Kim, J.-E. 2020. Lipid accumulation inhibition and lipid oxidation increase of fermented white sword bean extracts in 3T3-L1 adipocytes. Master’s degree thesis, Hanyang University, Korea, pp. 1–50.

## Slide 2
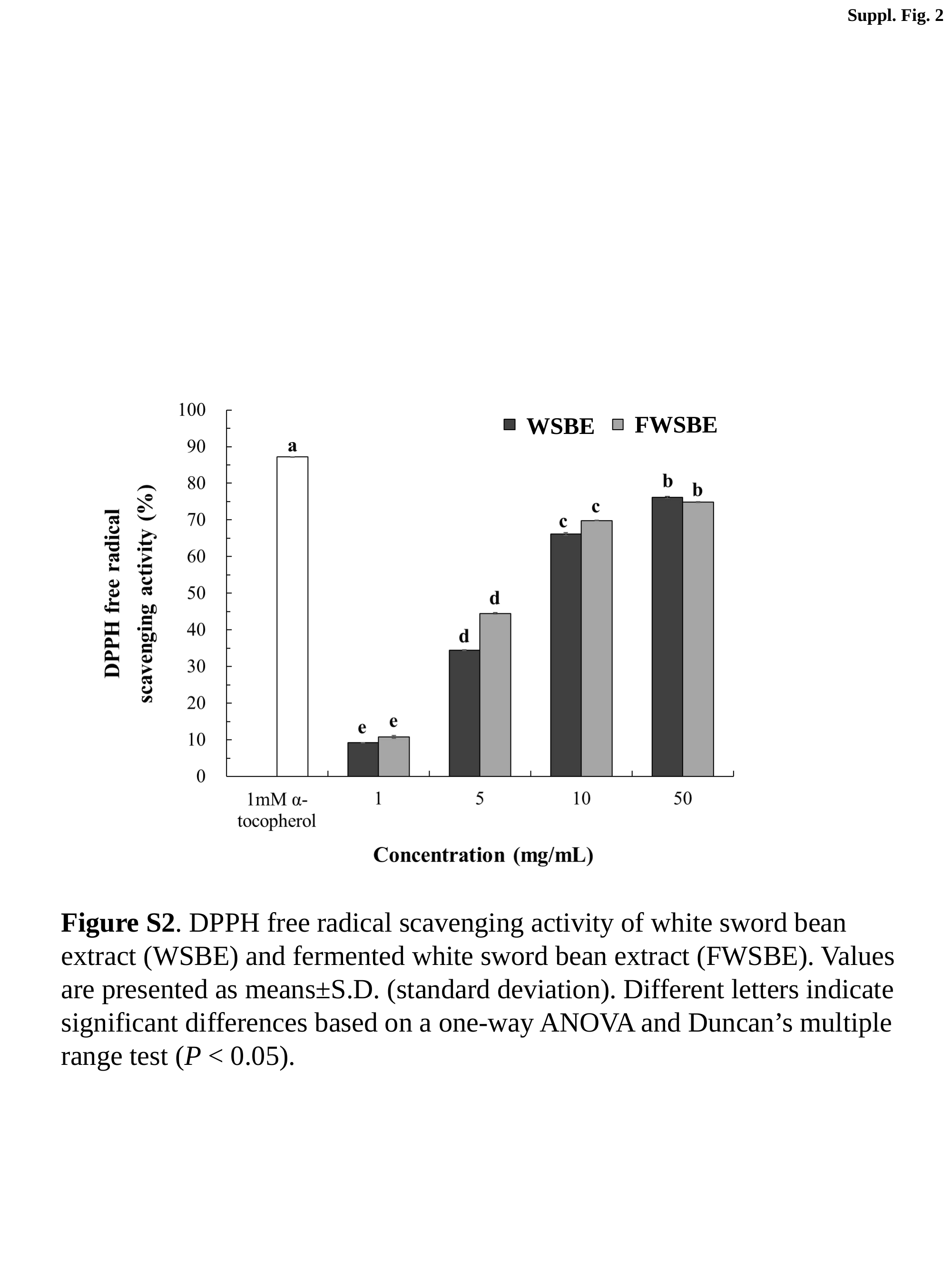

Suppl. Fig. 2
FWSBE
WSBE
Figure S2. DPPH free radical scavenging activity of white sword bean extract (WSBE) and fermented white sword bean extract (FWSBE). Values are presented as means±S.D. (standard deviation). Different letters indicate significant differences based on a one-way ANOVA and Duncan’s multiple range test (P < 0.05).
